# Differential Gene Expression Associated with Idiopathic Epilepsy in Belgian Shepherd Dogs

**DOI:** 10.3390/genes15111474

**Published:** 2024-11-15

**Authors:** Nathan Kinsey, Janelle M. Belanger, Anita M. Oberbauer

**Affiliations:** Department of Animal Science, University of California, Davis, CA 95616, USA; natkinsey@ucdavis.edu (N.K.); jmbelanger@ucdavis.edu (J.M.B.)

**Keywords:** idiopathic epilepsy, gene expression, seizure, Belgian shepherd, dog, interferon signaling

## Abstract

Background: Idiopathic epilepsy (IE) disproportionately affects Belgian shepherd dogs and although genomic risk markers have been identified previously in the breed, causative variants have not been described. Methods: The current study analyzed differences in whole blood RNA expression associated with IE and with a previously identified IE risk haplotype on canine chromosome (CFA) 14 using a transcriptomics RNA-seq approach. Results: *MFSD2A* and a likely pseudogene of *RPL19*, both of which are genes implicated in seizure activity, were upregulated in dogs with IE. Genes in the interferon signaling pathway were downregulated in Belgian shepherds with IE. The CFA14 risk haplotype was associated with upregulation of *CLIC1*, *ACE2*, and *PIGN* and downregulation of *EPDR1*, all known to be involved with epilepsy or the Wnt/β-catenin signaling pathway. Conclusions: These results highlight the value of assessing gene expression in canine IE research to uncover genomic contributory factors.

## 1. Introduction

In the domestic dog (Canis lupus familiaris), one of the most common neurological disorders is epilepsy [1]. Epilepsy, defined as repeated seizures, is stressful for both dog and owner and, if symptoms are severe, can lead to euthanasia [2,3]. The expense of diagnosis places a large burden on dog owners and treatment options may cause dangerous side effects while frequently failing to control seizures [4]. Idiopathic epilepsy (IE), a form of epilepsy characterized by genetic inheritance, is observed in most, if not all, dog breeds with certain breeds having a higher incidence than average [5]. Relative to other dog breeds, the prevalence of IE is considered elevated in the Belgian Sheepdog (BS) and Belgian Tervuren (BT), with estimates of 7.5% in both [6,7].

Genome-wide association studies (GWAS) have been undertaken in various dog breeds, including the Dutch Partridge Dog [8], Petit Basset Griffon vendéen [9], Irish Wolfhound [10], and Belgian Shepherd [11,12], with the goal of identifying the underlying genetic cause of IE. The relatively high prevalence of IE in Belgian Shepherds has led investigators to focus efforts on BS and BT dogs. Those studies have revealed risk regions on canine chromosome (CFA) 14 [12] and CFA 37 [13]. On CFA 14, an ACTG risk haplotype was identified near *RAPGEF5*, a gene proposed to have neurological function and whose downregulation has been associated with focal seizures in rats [14,15]. The ACTG haplotype is associated with a 3-base pair insertion in exon 1 of the *RAPGEF5* gene and has been shown to disrupt cellular localization [16,17]. The regions identified on CFA 37 are near *ADAM23* [18,19] and *KLF7* [20,21,22], genes implicated in canine epilepsy and neuronal development, respectively, although causative variant(s) have not been identified. Despite the identification of these risk loci, they only account for a small proportion of IE risk in dogs. A transcriptomics approach may reveal altered expression profiles reflecting underlying genetic changes leading to the discovery of additional mutations associated with canine IE. Using transcriptomics to profile the expression patterns of the genome has facilitated characterizing the impacts of many genetic variants simultaneously in other neurological conditions such as autism spectrum disorder [23]. Furthermore, functional impacts of genetic variants in intergenic regions and non-coding RNAs, including microRNA (miRNA), short interfering RNA (siRNA), and long non-coding RNA (lncRNA), can also be assessed through transcriptomic analyses [24,25].

The present study used transcriptomics to identify risk loci contributing to IE in the Belgian Shepherd. Gene expression analysis of Belgian Shepherds was undertaken to determine genes altered by IE status and if the risk haplotype on CFA 14, previously identified by GWAS, was associated with altered RNA expression thereby defining a functional impact of that risk variant. The findings provided insight into metabolic pathways that may underly IE in the dog.

## 2. Results

### 2.1. Differential RNA-Seq Expression Analysis

**Figure 1:**
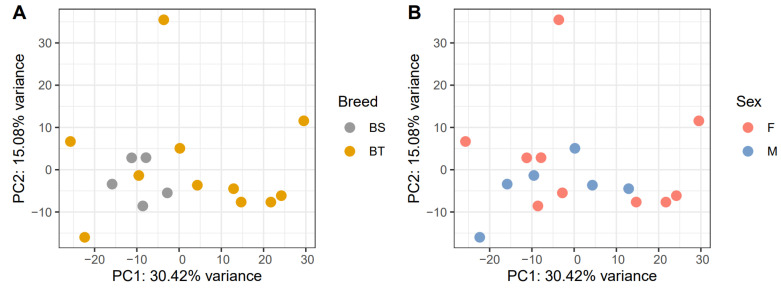
Principal component analysis of gene abundance comparing breed variety (**A**) and sex (**B**). Gene abundance estimates for 16 Belgian Shepherds were plotted along their first two principal components (PC1, horizontal axis, and PC2, vertical axis). Clustering was determined by visual assessment.

Gene abundance estimates plotted by PCA show the BS dogs clustering more closely than the BT dogs along the first two principal components (Figure 1). Sex did not appear to cluster in this way. To account for breed variety’s correlation with the principal components, it was included as a covariate in the differential expression models [26] while sex was not.

Ten genes had significant differential expression (FDR-corrected *p*-value < 0.10) in the blood samples of IE cases versus healthy controls, with two being upregulated and eight being downregulated in IE cases (Table 1). The upregulated genes were *ENSCAFG00000004857* and *MFSD2A* with 7.6- and 1.6-fold increased expression in IE cases, respectively. Genes that were downregulated included the novel gene *ENSCAFG00000002440*, exhibiting a 20-fold reduction in expression, *MAST4* with a 3.5-fold reduction, and six others with smaller observed reductions in dogs with IE. A pathway enrichment analysis of the differentially expressed genes revealed that the interferon (IFN) signaling pathway was overrepresented (FDR-corrected *p*-value < 0.01). Specifically, five of the downregulated genes, *HERC5*, *HERC6*, *RSAD2*, *DDX58*, and *IFI44*, all participate in IFN signaling [27], several of which, *HERC5*, *HERC6*, and *IFI44*, have been implicated in seizures and neurological development [28,29]. Genes associated with the previously identified risk loci on CFA 14 and CFA 37, that is *RAPGEF5*, *ADAM23*, and *KLF7*, did not demonstrate differential expression between IE cases and healthy controls (Table 1). The full gene expression data from both Kallisto and Salmon models are presented in an additional file (Table S1).

When only considering dogs with and without the risk genotype, five genes were upregulated and one was downregulated (FDR-corrected *p*-value < 0.10) in dogs with the ACTG risk haplotype when compared to dogs without the haplotype (Table 2). The full gene expression data from the haplotype analysis are presented in an additional file (Table S2). The expression of *ADAM23*, *KLF7*, and *RAPGEF5*, the three genes previously implicated in IE risk, was unchanged in dogs with and without the ACTG risk haplotype.

### 2.2. RT-qPCR Validation

To reinforce the RNA-seq results, a subset of the three differentially expressed genes, *HERC5*, *IFI44*, and *MFSD2A*, as well as *RAPGEF5* were selected for RT-qPCR validation in a different cohort of dogs. Genes involved in the IFN pathway that were selected for confirmation were those specifically implicated as having a role in neurological function. *HERC5* was selected because two indicated genes from the *HERC* family were found to be downregulated in the present study and large and small HERC proteins are directly associated with seizures and neurological development, respectively [29]. *IFI44* was selected due to its association with febrile seizures in humans [28] and *MFSD2A* was selected for its role in brain development and blood-brain barrier function [30,31]. All three selected genes had a mean fold change expression that matched the direction of regulation indicated by the RNA-seq expression analysis (Figure S1). Specifically, *HERC5* and *IFI44* demonstrated downregulation, whereas *MFSD2A* demonstrated upregulation. The expression pattern of *RAPGEF5* did not exhibit high amounts of differential expression (only a slight upregulation) between IE cases and healthy controls, again validating the RNA-seq observation.

### 2.3. Whole Genome Sequencing

Using the differentially expressed genes to drive the investigation of potential IE causal variants in the whole genome sequence, the 1 Mb regions surrounding the top ten differentially expressed genes were found to have a total of 305 different sequence variants (*p* < 0.01) between IE cases and healthy controls (Table 3). While a majority of those variants do not have a predicted impact on protein function or gene regulation (Table S3), twelve variants are purported to affect regions that control gene expression in humans. There were no impactful variants detected in the regions associated with genes differentially expressed in dogs having the ACTG risk haplotype.

In a splice region for *MFSD2A* (CFA15: 3226673), five of the controls were homozygous for a single nucleotide deletion, with the remaining two controls being heterozygous. The IE dogs, in contrast, had numerous homozygous reference genotypes (50%), a moderate number of heterozygotes (38%), and a single dog homozygous for the deletion. In the first intron of *GAB2*, a gene near *ENSCAFG00000004857*, all the IE cases were heterozygous or homozygous for a 5-bp deletion (CFA21: 20310516), while only two of the healthy controls were heterozygous for the variant, and the remainder were homozygous reference (75%). A single nucleotide deletion in an intergenic region near both *HERC5* and *HERC6* (CFA32: 30585125) was disproportionally prevalent in IE cases (66% homozygous and 33% heterozygous) and seen only as heterozygotes in healthy controls. In a 400,000 bp intergenic region upstream of *RSAD2*, nine variants were heterozygous in all but one IE case (89%) and homozygous reference in all healthy controls (Table S3). Additionally, two variants (CFA17: 4413952 and CFA17: 4413996) have a single-nucleotide substitution in the 5′ UTR of *RSAD2*, creating premature start codons; these were observed in all but one of the IE cases (22% homozygous for the substitution and 66% heterozygous), with only one of the healthy controls heterozygous (12.5%) and the remainder homozygous reference.

## 3. Discussion

In this study, transcriptomics expression analysis identified genes with significantly differential expression between healthy and IE dogs. Additionally, genes were differentially regulated in Belgian Shepherd dogs having the CFA 14 ACTG haplotype shown to be associated with IE risk [12]. As the present study is the first to investigate differential mRNA expression in IE dogs, these results suggest novel gene associations for canine epilepsy, including genes involved in immune signaling and neural development. Many of the genes exhibiting differential expression have been shown in previous human and mouse studies to be associated with seizures [31,32,33,34], epilepsy [35], and neurological development [30].

Two genes, *ENSCAFG00000004857* and *MFSD2A*, exhibited increased expression in IE cases when compared to controls. A BLAST of the *ENSCAFG00000004857* sequence suggests that it is likely a pseudogene of *RPL19* [36], a ribosomal protein [37] that has elevated expression in epileptic rat models [37]. Additionally, *ENSCAFG00000004857* lies within an intron of *INTS4*. No variants that could account for the differential expression were found when comparing IE cases and controls in *INTS4* or in *ENSCAFG00000004857*, suggesting the existence of variants upstream of the genes themselves. The upregulation of *MFSD2A* in IE dogs warrants further investigation due to its established role in the development of the blood-brain barrier in mice [30]. While the increased expression was modest, with a log-fold change of 1.59, disruptions to proper molecular transport across this barrier have been associated with neurological disorders such as Alzheimer’s and Parkinson’s disease [38]. Furthermore, *MFSD2A* gene mutations in humans have been associated with microcephaly, a disease that alters brain size and can result in seizures [31,32]. The splice region variant in *MFSD2A* detected in the present study provides a potential explanation of its impact on the gene’s differential regulation, as splice region variants are known to affect gene expression [39], although the genotypic frequency of the splice variant between IE dogs and controls does not suggest that it is a major factor of IE.

Pathway analyses revealed that the IFN signaling pathway was significantly enriched by differentially expressed genes in IE dogs, suggesting a link between epilepsy and immune function. The elevated expression of genes associated with the immune system may reflect the direct risk for IE. For instance, dysfunctional HERC proteins that participate in IFN signaling have been shown to cause Purkinje cell death and deficits in neurotransmission [29]. Alternatively, the elevated expression may reflect the sequelae of seizure activity and the body’s adaptive response.

**Figure 2:**
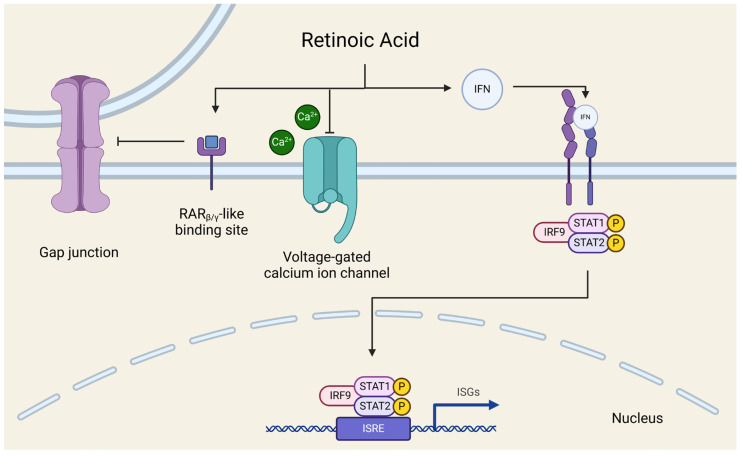
The impact of retinoic acid on neuronal activity and the IFN signaling pathway [50].

A great many studies have identified a role for the immune system in epileptogenesis [40,41] including canine IE [42]. This is exemplified by the fact that immunoglobulin therapy has been shown to have anticonvulsant effects for several seizure disorders [43]. The present study supports previous canine proteomic profiling which indicates that immunity proteins are the most impacted pathway in canine epilepsy [44]. Given that many of the downregulated genes identified in this study participate in the IFN pathway, this suggests that there may be a high-level inhibition of the pathway impacting multiple signaling steps. In addition to its activation in response to viral infection [45], the IFN signaling pathway is triggered by lipopolysaccharides, retinoic acid (RA), and genotoxic substances [46]. The RA trigger is of particular interest due to its antiepileptogenic properties [47]; if the retinoid metabolic pathway was disrupted, it may both reduce activation of the IFN signaling pathway and increase seizure activity (Figure 2). A BLAST of pseudogene, *ENSCAFG00000002440* indicates a high synteny to *AKR1B1* in multiple species [36]. *AKR1B1* may contribute to the regulation of retinoic acid, and a downregulation of *ENSCAFG00000002440* as observed in the present study could disrupt retinoid metabolism by altering the regulation of retinoid receptors [48]. Retinols are thought to lessen seizure activity through their inhibition of gap junction and synaptic signaling and voltage-gated calcium ion channels [47]. Importantly, *AKR1B1* is duplicated in the dog genome relative to the wolf genome and has a suggested role in domestication, with the duplicated version assuming new functional roles in fatty acid synthesis and managing oxidation [49].

Retinoic acid increases extracellular IFN secretion [51,52], and IFN then binds to receptors to activate a complex of signal transducer and activator of transcription (STAT) proteins and interferon regulatory factor (IRF) [46]. This complex induces transcription at interferon-sensitive response element (ISRE) promoter regions that regulate the expression of interferon-stimulated genes (ISGs) [46]. Aside from participating in IFN signaling, RA has multiple mechanisms of controlling cellular communication in neurons that reduce epileptogenesis [47]. Specifically, RA interacts with neuronal G proteins, which can inhibit calcium flow across voltage-gated calcium ion channels, altering neurotransmitter release [53]. Additionally, RA binds with retinoic acid receptor (RAR)-like binding sites, signaling gap junction connexins to close [54] thereby decreasing seizure activity [55].

Pathway analysis did not detect a connection among the five genes upregulated in dogs with the ACTG risk haplotype. One upregulated gene, *CLIC1*, encodes a chloride ion channel. This is a promising candidate for IE as the misregulation of chloride homeostasis has been shown to affect the GABAergic neurotransmission signaling in the brain, which can lead to temporal lobe epilepsy in humans [56,57,58]. Additionally, the upregulated genes, *CLIC1* and *ACE2*, and the downregulated gene, *EPDR1*, have been shown to participate in the Wnt/β-catenin signaling [59,60,61], a pathway that may help to control seizure activity [62] and is the pathway potentially affected by the *RAPGEF5* insertion variant associated with the ACTG risk variant [17]. Notwithstanding that *RAPGEF5* expression was unaltered in dogs having the ACTG risk haplotype, an alteration in the expression of other genes might be an attempt to compensate for a reduction in the functionality of the RAPGEF5 protein. Another gene upregulated, albeit minimally altered in expression, the role of *PIGN* is in anchoring proteins to blood cells [63], and mutations in the gene have been linked to various epilepsy phenotypes including generalized seizures in children [64]. The two remaining upregulated genes associated with the ACTG risk haplotype, *RHEX* and *SLC45A3*, do not have a clear connection to either epilepsy or the Wnt/β-catenin signaling pathway.

The majority of the differentially expressed genes associated with the IE phenotype were downregulated. Pathway analysis identified the IFN signaling pathway as enriched with the genes significantly downregulated in IE dogs. The genetic variants identified in the whole genome sequence found near the differentially expressed genes could affect the expression of genes with IFN and neurological function. Three of the five genes downregulated in this way, *HERC5*, *HERC6*, and *IFI44*, have been previously associated with neurological development and seizures [28,29]. The variants found near *HERC5* and *HERC6* lie in a region associated with a locus known in humans to regulate the expression of *XBP1* [65]. Alterations in *XBP1* expression are implicated in neurological disorders including epilepsy [66,67]. Additionally, *XBP1* is involved in immune cell development and differentiation, including direct binding to IFN promoter regions [68]. The variant nearest the upregulated *ENSCAFG00000004857* is an insertion in the first intron of *GAB2* and is predicted to be a proximal enhancer in humans [65]. Disrupting a potential *GAB2* enhancer may affect IFN signaling by modulating the expression of GAB2, a protein known to compete for binding sites on IFN receptors [69]. Additionally, *GAB2* has known associations with seizures, and is downregulated in rats with temporal lobe epilepsy [70]. Although in the present study, *ENSCAFG00000004857* expression was elevated, expression of GAB2 was not determined as being elevated or reduced in the IE dogs. The *GAB2* insertion is near the adjacent gene *NARS2*, which has often been associated with epilepsies in human infants and children [71,72,73]. Of note is that the region adjacent to *ENSCAFG00000004857* is annotated by NCBI as *NARS2* and *GAB2*, although the Ensembl annotation of Dog10K Boxer Tasha represents it as one gene, *NARS2* [36,74]. Both genes have been implicated in epilepsy and the variants identified coupled with the altered gene expression offer clues into the mechanism of IE development.

The nine variants identified upstream of *RSAD2* occur in a region known to regulate human expression of *KLF6*, *ELF3*, and *SPDEF* [65] and thus may play a role in both IFN gene regulation and neurology by affecting these regulatory factors. *KLF6* is a paralog of *KLF7*, a gene previously associated with canine IE and neural development [20,21,22]. *ELF3* and *SPDEF* have been implicated in the upregulation of the IFN regulatory factor 6 [75] and inhibit gene expression in the IFN pathway [76], respectively. These variants may serve to connect IFN pathway regulation observed in the present study and the underlying neurology of IE.

The most downregulated gene in IE cases, *ENSCAFG00000002440*, is a pseudogene with high synteny to *AKR1B1* [74], a gene known to regulate retinoid acid levels [48]. Additionally, *AKR1B1* has been shown to induce prostaglandin PGF2α when signaled by COX1/COX2 [77]. It is possible that *ENSCAFG00000002440* may share a similar function. While multiple studies have implicated the COX/prostaglandin pathway in epilepsies, they suggest that PGF2α protects against seizure activity [33] and deficient PGF2α increases seizure susceptibility [34]. Given this, the downregulation of a gene acting similarly to *AKR1B1* could be expected to increase seizure risk, as suggested by the observed association in the present study, although there were no identified variants within *ENSCAFG00000002440* that could account for the downregulation. Furthermore, despite *ENSCAFG00000002440* being 3.9 MB away from the previously identified *RAPGEF5* IE risk markers, there is no clear evidence that these loci interact; genomic regions have not been observed to influence expression if greater than 2–3 MB away [78]. Future investigation may seek to confirm if *ENSCAFG00000002440* participates in the same pathway that *AKR1B1* induces.

As this study used mRNA to assess genomic expression, future work may determine if there is differential expression of regulatory RNAs in IE dogs, including miRNA, siRNA, and lncRNA. This may help characterize genetic variants previously associated with IE, as well as describe the mechanisms for differential gene expression identified in this study. Furthermore, while the differentially expressed genes in whole blood significantly associate IE with immune function in the Belgian Shepherd, the present study may be limited by the sample source. In this study, RNA was extracted from blood due to the impracticality of collecting brain tissue samples including which neural tissue to use and temporal timing of collection. Despite blood effectively serving as a proxy tissue for identifying differentially expressed genes in human epilepsies [79,80], the results may not fully reflect the variety of expression differences between IE and healthy dogs in all tissue types. Similarly, genes previously associated with IE in Belgian Shepherds such as *RAPGEF5*, *KLF7*, and *ADAM23* may have altered expression in IE dogs that can only be observed in neural tissues, despite their expression in canine blood [81], or these previously associated genes may not have altered transcription but their impacts may be due to changed localization [17] or protein structure. However, it is also possible that the observed IE-associated expression differences would not be detectable in many other tissues, reaffirming the utility of blood samples in epilepsy research.

The observed expression differences are valuable for understanding mechanisms associated with IE and reaffirm the role of the immune system in canine IE. The downregulation of particular genes in the immune pathway offer targets for further research to determine a causal relationship and if the altered expression pattern increases seizure risk or if these genes are a byproduct of past seizure activity. Future work may monitor gene regulation in IE individuals over time to differentiate changes in expression resulting from repeated seizures. This is especially relevant given the presence of variants that have the predicted role of altering gene expression with putative roles in epileptogenesis. Further research may also explore if the observed downregulation of IFN signaling is due to a link between IE and the pathway itself or one of the pathway precursors such as retinoic acid.

## 4. Materials and Methods

### 4.1. RNA Preparation and Sequencing

Dogs defined as having IE (n = 9, cases) had multiple generalized seizures with disease onset after one year of age (mean 4.63 years old). Sample phenotypes were classified based on previously described criteria [12] and in brief, IE diagnosis followed guidelines set by the International Veterinary Epilepsy Task Force for a Tier I confidence level diagnosis [82]. This includes an unremarkable veterinary examination of neurology, blood, and urine in IE dogs. Healthy control dogs (n = 7, controls) were at least 7 years of age (mean 10.47 years old) with no reported health issues. While dogs within phenotype groups (IE and healthy) were unrelated at the grandparent level, relatedness was allowed across phenotype groups. Dogs were genotyped for the CFA 14 risk haplotype to ensure a relative balance of breed variety across all haplotypes (Table 4). Two additional dogs with focal seizures were included in the risk haplotype analysis that were not present in the generalized IE analysis. Power calculations were performed using the Scotty web tool and default parameters [83] to determine that five replicates per treatment group were sufficient to detect differential gene expression between the affected and healthy groups. Moreover, recently published studies in dogs have successfully assessed differential gene expression using similar or fewer replicates per study group [84,85].

RNA-seq analyses of blood have been used to study genomic expression in human epilepsies revealing mutations associated with IE [79,80]. Whole blood was collected from 16 Belgian shepherds (5 BS = 4 F, 1 M; 11 BT = 6 F, 5 M) in DNA/RNA shield blood collection tubes, which can keep RNA stable for up to 5 days at room temperature (Zymo Research, Irvine, CA, USA), and stored at −80 °C. Unprocessed whole blood samples were then sent to Novogene (Beijing, China) in the collection tubes for mRNA extraction and sequencing. Globin mRNA was depleted from the blood using GLOBINclear (Invitrogen, Waltham, MA, USA) to improve exonic coverage. PolyA capture was used to isolate mRNA which was then randomly fragmented and primed. Second-strand cDNA was marked to enable directional sequencing following adenylation and adapter ligation [86]. Fragments with an insert size of 150 bases were selected for sequencing. The extracted mRNA was then sequenced using the NovaSeq PE150 platform (Illumina, San Diego, CA, USA). Sequencing quality was validated with MultiQC (1.10.1) and FastQC (0.11.9) software to assess base sequence quality scores, GC content distribution, and sequence duplication and overrepresentation [87,88]. Adapter sequences were trimmed from sequence reads and bases with a quality score below 2 were trimmed with a sliding window of four bases using Trimmomatic (0.39) [89].

### 4.2. Read Quantification and Differential Expression Analysis

Ensembl’s transcriptome of the Dog10K Boxer Tasha genome assembly was used as a reference for quantification [74,90]. Transcript quantification was performed on the trimmed reads using both Kallisto (0.46.2) and Salmon (1.3.0) [91,92]. Kallisto was run using default parameters and Salmon was run using the sequence and GC bias correcting options, which is recommended for improving accuracy in differential expression analyses [92]. Kallisto and Salmon yield concordant results with the advantage that Kallisto is more rapid and has better performance with correlation statistics [93]. The transcript quantification estimates were summarized to the gene level using the tximport (1.26.1) and GenomicFeatures (1.50.4) R packages [94,95,96] with default parameters. Principal component analysis (PCA) was performed on the gene abundance estimates using the DESeq2 R package to determine if breed variety or sex were responsible for any large variation in the data, and if so, these variables could be accounted for in the modeling step [94,97]. Two different differential expression models were built in DESeq2 (1.38.3) comparing expression differences between healthy controls and IE cases as well as dogs with the ACTG risk haplotype and dogs without the ACTG risk haplotype [98,99,100]. Models included covariates that were identified by PCA as contributing to a large amount of variation in the data. The models performed a Wald test with the null hypothesis assuming that the log fold change between treatment groups was equal to zero. A correction for false discovery rate (FDR) was performed using the Benjamini–Hochberg method as previously described [101] for all genes that have expression in blood, accounting for false positive genes that are not truly differentially expressed between groups. Significance was defined as having a corrected *p*-value less than 0.10 [102,103]; this permissive cutoff was chosen to maximize the genes used for pathway analysis, while avoiding a high FDR with the additional correction outlined below.

Genes with statistically significant gene expression between IE cases and controls were compiled for pathway analysis using the Reactome (90) web tool to identify enriched biological pathways [27]. For each pathway indicated by these genes, a binomial test was performed with the null hypothesis assuming a random distribution of genes across known pathways. A correction for FDR was performed for each known pathway using the Benjamini–Hochberg method [101], accounting for false positive pathways that are not truly enriched by differentially expressed genes. Significance for pathway analysis was defined as having a corrected *p*-value less than 0.05.

### 4.3. Reverse Transcription Quantitative Polymerase Chain Reaction (RT-qPCR)

Genes that were expressed at significantly differential levels were chosen for RT-qPCR confirmation in a separate cohort of 10 Belgian shepherds (5 IE = 2 BS, 3 BT; 5 healthy = 2 BS, 3 BT). Dogs were classified as healthy controls or IE cases and whole blood was collected as described above. Whole blood samples were sent to the UC Davis Genome Center for RNA extraction and RT-qPCR as previously described [104]. Specifically, primers and TaqMan probes (Table S4) were designed for target genes and reference genes *HPRT1* [105] and *GAPDH* [106] using Primer Express software (3.0.1) (Thermo Fisher Scientific, Carlsbad, CA, USA). The qPCR systems were validated in triplicate and standard curves were plotted against 10-fold dilutions of DNA positive for target genes. The slope of the curve was used to ensure that all target genes had amplification efficiencies greater than 90%.

RNA extraction was performed using a Quick-RNA whole blood kit, following manufacturer protocols (Zymo Research, Irvine, CA, USA). Each PCR reaction contained 400 nM of each primer, 80 nM TaqMan probe, TaqMan Universal PCR Mastermix (Thermo Fisher Scientific, Carlsbad, CA, USA), and 5 μL diluted cDNA. Reactions were run in triplicate using an ABI PRISM 7900 HTA FAST automated fluorometer, using standard conditions (Thermo Fisher Scientific, Carlsbad, CA, USA). Gene expression was quantified using the comparative CT method, averaging the two reference genes to normalize reference gene CT values [107]. Any concerns regarding the sample size of the RNA-seq analyses were minimized by the confirmation of expression profiles in a different cohort of dogs.

### 4.4. DNA Preparation and Whole Genome Sequencing

Whole blood was previously collected by our laboratory for 17 Belgian Tervuren (8 healthy = 3 F, 5 M; 9 IE = 4 F, 5 M) and DNA was extracted using the Qiagen DNA Blood Mini Kit, following manufacturer protocols (Qiagen Inc., Valencia, CA, USA). Extracted DNA was sent to Novogene (Beijing, China) for polymerase chain reaction (PCR)-free library preparation and sequencing using the NovaSeq PE150 platform (Illumina, San Diego, CA, USA). FASTQ files collected from different lanes of the same sample were merged using the “cat” BASH command. Sequence reads were assessed for quality and trimmed using MultiQC, FastQC, and Trimmomatic as described above [87,88,89].

Trimmed reads were mapped to the Dog10K Boxer Tasha genome assembly [90] using the Burrows–Wheeler Alignment tool (0.7.17) with default parameters [108]. The resulting mapped read files were then sorted and indexed using SAMtools software (1.14) [109]. While PCR duplicates are not a concern with PCR-free library preparation, optical duplicates were removed using the Picard toolkit (2.26.11) [110]. Deduplicated reads were then used for variant calling using the short haplotype caller FreeBayes (1.3.4) [111]. To comply with computational limits, regions with greater than 1000× coverage were skipped by the variant caller.

Variants within 1 Mb of differentially expressed genes were extracted using BCFtools (1.19) [109]. To identify variants that significantly differ in these regions between healthy and IE dogs, a Fisher’s exact test using a dominant model was performed by SnpSift (5.2c) and filtered with a *p*-value cutoff of 0.05 [112]. The functional effect of these variants were predicted with SnpEff software (5.2c) [113] using the Ensembl (112) annotation of the Dog10K Boxer Tasha reference assembly [74]. The variant coordinates were lifted over to the human GRCh38 reference assembly [114] using the UCSC liftOver web tool [115]. The ReMap database (4) was accessed through the UCSC genome browser and was used to determine if variants were located in known regulatory regions in humans [65,115].

## Figures and Tables

**Table 1:** Differentially expressed genes between IE cases and healthy controls. The top ten differentially expressed genes from the Kallisto and DESeq2 analysis and three genes previously associated with canine IE.

Gene	Log_2_ Fold Change *	FDR-Corrected *p*-Value
*ENSCAFG00000004857*	7.60	3.64 × 10^−2^
*MFSD2A*	1.59	6.43 × 10^−2^
*HERC6*	−1.33	5.71 × 10^−2^
*EPSTI1*	−1.37	7.52 × 10^−2^
*DDX58*	−1.45	9.60 × 10^−2^
*HERC5*	−1.53	3.06 × 10^−2^
*IFI44*	−1.58	2.93 × 10^−2^
*RSAD2*	−2.13	6.63 × 10^−2^
*MAST4*	−3.50	7.26 × 10^−3^
*ENSCAFG00000002440*	−20.70	3.63 × 10^−7^
*ADAM23*	−3.45	9.99 × 10^−1^
*KLF7*	0.21	9.99 × 10^−1^
*RAPGEF5*	0.10	9.99 × 10^−1^

* Negative log_2_ fold change values indicate downregulation, and positive values indicate upregulation in IE dogs.

**Table 2:** Differentially expressed genes between dogs with and without the ACTG risk haplotype. The top six differentially expressed genes from the Kallisto and DESeq2 analysis and the three genes previously associated with canine IE.

Gene	Log_2_ Fold Change *	FDR-Corrected *p*-Value
*CLIC1*	18.85	1.70 × 10^−3^
*RHEX*	3.51	1.80 × 10^−3^
*ACE2*	6.44	2.41 × 10^−2^
*PIGN*	0.81	2.41 × 10^−2^
*SLC45A3*	4.62	4.57 × 10^−2^
*EPDR1*	−1.37	9.55 × 10^−2^
*ADAM23*	−3.01	9.98 × 10^−1^
*KLF7*	0.07	9.98 × 10^−1^
*RAPGEF5*	0.16	9.98 × 10^−1^

* Negative log_2_ fold change values indicate downregulation in ACTG dogs while positive values indicate upregulation.

**Table 3:** Whole genome sequence variants near differentially expressed genes comparing IE cases and healthy controls. The analyzed region is specified in coordinates from the Dog10K Boxer Tasha genome assembly. Of variants within 1 MB of a differentially expressed gene, Fisher’s exact test using a dominant model showed differences between IE case and control variant genotypes (*p* < 0.05).

CFA	Gene	Position (bp)	Number of Significant Variants *p* < 0.05	Number of Variants Within Gene
21	*ENSCAFG00000004857*	20,281,342–21,282,239	281	0
15	*MFSD2A*	2,723,565–3,736,071	11	1
32	*HERC6*	29,703,843–30,759,559	109	6
22	*EPSTI1*	7,354,646–8,600,477	22	0
11	*DDX58*	47,886,818–48,922,130	33	0
32	*HERC5*	29,653,835–30,697,247	85	0
6	*IFI44*	70,521,268–71,559,185	51	4
17	*RSAD2*	3,913,918–4,934,440	1524	84
2	*MAST4*	48,559,935–50,095,394	23	17
14	*ENSCAFG00000002440*	30,665,380–31,666,329	134	0

**Table 4:** Sample size breakdown of CFA 14 haplotypes in the studied population. The IE status and breed varieties of all haplotypes at the CFA 14 risk locus. The ACTG haplotype confers the highest IE risk compared to the other two haplotypes.

CFA 14 Haplotype	Epileptic	Control	BS	BT
ACTG	6	0	3	3
CTCT	3	5	2	6
CTCG	2	2	1	3

## Data Availability

The original contributions presented in the study are included in the article/Supplementary Materials, further inquiries can be directed to the corresponding author.

## Supplementary Materials

The following supporting information can be downloaded at: https://www.mdpi.com/article/10.3390/genes15111474/s1, Table S1: Genes differentially expressed in dogs with IE; Table S2: Genes differentially expressed in dogs with the ACTG risk haplotype; Table S3: Whole genome variants of genes differentially expressed in dogs with IE; Table S4: Primers and probes for target and reference genes; Figure S1: Box and whisker plot of RT-qPCR fold change expression for *HERC5*, *IFI44*, and *MFSD2A* genes in IE dogs when compared to controls.
