# Supplementary figures and images for: Differential Gene Expression Associated with Idiopathic Epilepsy in Belgian Shepherd Dogs
Source: Genes (Basel). 2024 Nov 15;15(11):1474. doi: 10.3390/genes15111474 (PMC11593353; doi:10.3390/genes15111474)

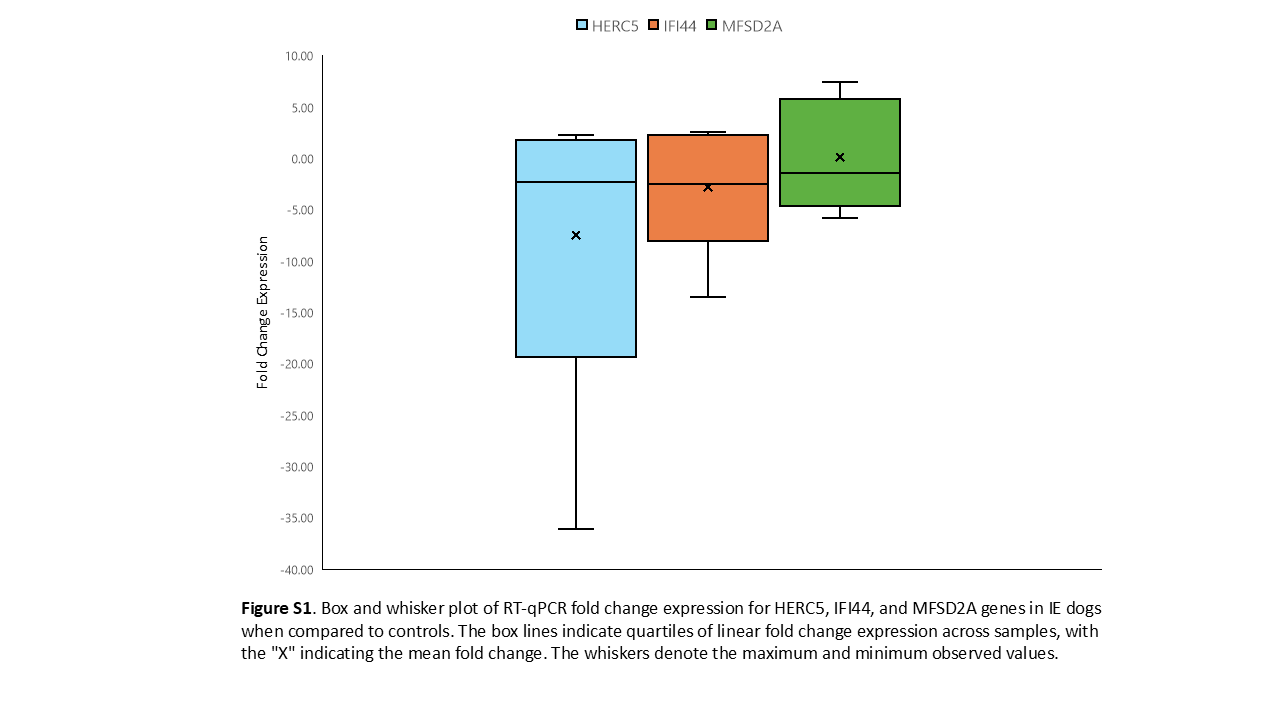

Supplement: Supplementary file 1 [file genes-15-01474-s001.zip › FigureS1.tif]
